# Facilitating Evidence-Based Practice among Nurses in a Tertiary General Hospital: A Six-Year Practice of an Implementation Strategy Informed by the i-PARIHS Framework

**DOI:** 10.1155/2024/8855667

**Published:** 2024-03-19

**Authors:** Yuping Zhang, Zhiting Guo, Shuangyan Xu, Meiqi Yao, Xiuqin Feng, Meijuan Lan, Jingfen Jin

**Affiliations:** Nursing Department, The Second Affiliated Hospital of Zhejiang University School of Medicine, Hangzhou, Zhejiang, China

## Abstract

**Aims:**

To develop an evidence-based practice (EBP) facilitating strategy informed by the Integrated Promoting Action on Research Implementation in Health Services (i-PARIHS) framework and evaluates the strategy's effectiveness on promoting EBP.

**Background:**

Nurses are increasingly expected to use research evidence in practice to improve patients' outcomes, but the application of such evidence is still unsatisfactory. The facilitation of EBP in nursing needs the organization and the individual levels.

**Methods:**

Based on an analysis of EBP-promoting strategies and expert consultation, the core team of the nursing department developed an EBP group-based and project-oriented strategy under the guidance of the i-PARIHS framework. The strategy was implemented for six years and evaluated by a longitudinal design. The validated Chinese version of the self-reported Evidence Based Practice Questionnaire (EBPQ) was used to assess the attitudes, knowledge, and practice of the nurses.

**Results:**

The mean score of the total EBPQ was 4.46 (SD = 0.92) after one year of the EBP strategy, and the scores for knowledge, attitudes, and practice of EBP members were higher than those of general nurses (*p* < 0.05). After four years, EBP members' scores continued to increase. EBP groups conducted 51 EBP projects in the areas of emergency nursing, surgical and medical nursing, critical care, and so forth. Of these, 33 projects improved nursing practice by changing nursing procedures, upgrading nursing tools, and developing nursing standards, resulting in improved patient outcomes.

**Conclusion:**

The EBP group-based and project-oriented strategy can promote EBP. The composition of the EBP group, the annual audit of EBP projects, and the roles of internal and external facilitators were key components of this EBP-facilitating strategy. *Implications for Nursing Management*. The strategy is an organizational-level EBP-facilitation approach which can improve nurses' academic preparation for EBP through ongoing training and sustained EBP behavior that involves annual projects.

## 1. Background

Evidence-based practice (EBP) involves integrating the current best evidence with clinical expertise, information on patient preferences, and available resources into the clinical decision-making process [1, 2], and it is critical to achieving optimal patient outcomes at the lowest possible cost [3]. As one of the important groups of practitioners in health care, nurses are increasingly expected to translate research evidence into practice to improve patients' outcomes [4–6]. Considerable research, resulting in the development of various theories/frameworks, policy, and funding as related to EBP, has been conducted [7–10]. Recent reviews show that education programs, reminders, and the use of champions help to ensure that nurses are aware of and use research evidence in clinical practice [11–13]. Nevertheless, applying the best research evidence into practice remains challenging. Further, there is limited understanding regarding which facilitation approaches are the most effective in enhancing evidence-based practice in certain contexts. [14].

Due to the dynamics and variability of evidence, complexity of the clinical environment, and differences in the capabilities of nurses, the implementation of EBP is still unsatisfactory, especially in low- and middle-income countries. Researchers have identified barriers to implementing evidence-based practice [15, 16] that include individuals' lacking the necessary knowledge and skills as well as organizations' not having enough funding or institutional support for EBP, among others [15–18]. Thus, it is critical to find ways to enhance the integration of research evidence into nursing practice.

In China, researchers have conducted surveys on the attitudes, knowledge, and skills of registered nurses (RNs) as related to EBP [19, 20]. Most nurses had positive attitudes toward EBP but lacked sufficient knowledge and skills for implementation [20, 21]. Despite the considerable effort taken to implement EBP in China and an increase in the number of EBP projects since 2013 [22, 23], there is an urgent need for EBP resources, increased support from clinical management, and collaboration between academic and clinical institutions. Further, unlike the case of physicians who have greater EBP knowledge [24] and hold more authority in medical domains, the focus of EBP implementation in nursing needs to be at the group or organization and individual levels [25]. Thus, our research aims to develop a strategy that includes the organizational aspect of facilitating EBP in a general hospital.

Facilitating EBP among nurses in a tertiary general hospital requires supportive evidence and a theoretical framework. The Promoting Action on Research Implementation in Health Services (PARIHS) framework is a multidimensional approach that posits that the successful implementation of evidence into practice is influenced by three key factors: the quality and type of evidence, characteristics of the setting or context, and the manner in which the evidence is introduced into or facilitates practice [26, 27]. PARIHS has been used extensively in a diverse range of settings and with various uses, including planning and delivering an intervention, data analysis, and the evaluation of study findings [26]. Nevertheless, the PARIHS framework has been criticized for lack of clarity in regard to its elements/subelements, definitions of key outcomes, and the relationships among elements/subelements [28]. The Integrated Promoting Action on Research Implementation in Health Services (i-PARIHS) framework was developed and refined by the PARIHS group and is now used in evidence-based projects globally [27]. The i-PARIHS framework addresses concerns about the conceptualization of as well as relationships and dynamics among the main framework elements, namely facilitation, innovation, recipients, and context [27].

We hypothesized that the i-PARIHS framework also is suitable to guide a program for facilitating EBP in our hospital. This hypothesis is based on the framework's comprehensive approach, which considers the complex interplay of evidence, context, and facilitation, an approach that is particularly relevant, given the unique challenges that nurses face. Based on the analysis of available knowledge translation strategies and characteristics of the hospital and nursing environment, we set up and implemented a group-based and project-oriented EBP strategy that has been sustained for six years. This study presents the development of this strategy and evaluates its effectiveness to shed light on the strategies and conditions that contribute to its success and limitations and to provide insight that can inform future efforts to promote EBP among nurses in similar settings.

## 2. Methods

### 2.1. Study Design

This study developed an EBP group-based and project-oriented strategy informed by the i-PARIHS framework and used a longitudinal design to observe the strategy's effectiveness. The framework was used to guide the development of the EBP-facilitating strategy at the hospital level. The strategy was practiced in a general hospital under the supervision of the nursing department for six years. The practice of the strategy from 2016 to 2019 was considered as the implementation phase. Also, year 2020–2021 was the sustained phase.

### 2.2. Setting and Participants

The study was conducted in a tertiary general hospital located in Hangzhou, Zhejiang Province, China. Because the hospital has a number of specialties, the Nursing Department was able to select six specialties which had more advantages and resources, and to set up EBP groups in 2016: Emergency and Intensive Care, General Surgery, Orthopedics, Neurology and Psychiatry, Gastroenterology and Respiratory Medicine, and Nursing Management. Nurses in these related departments who met the criteria could apply to participate in the study. Recruitment criteria included being committed to EBP, having certain skills related to EBP, priority will be given to candidates holding a master's degree or being a head nurse. A total of 34 nurses were recruited into six EBP groups based on these recruitment criteria. The groups were managed by the core team of the nursing department on an annual basis, and the number of groups was increased to 11, with 96 nurses, in 2021. The specialties of Neurosurgery, Intravenous Therapy, Operating Room, and Enteral Nutrition were added, and Emergency and Intensive Care were separated into two groups. The EBP group formation process and membership renewal are described in detail in the section of the EBP-facilitating strategy.  Table 1 shows the number of groups and nurses in each year.

### 2.3. EBP-Facilitating Strategy

A core team of the nursing department was set up to develop the EBP-facilitating strategy. The team included the director of nursing; vice director of nursing, who was in charge of nursing research and education; and three senior nurses who had received EBP training outside the hospital. The project was “Facilitating evidence-based nursing from the hospital level,” and the goal was to promote EBP for the nurses. Meetings of experts in the core team were conducted. Based on the analysis of EBP-promotion strategies and characteristics of the hospital and nursing environment, the team developed an EBP group-based and project-oriented strategy under the guidance of the i-PARIHS framework. In the framework, successful implementation involves facilitation of the innovation in coordination with the recipients in their local, organizational, and healthcare system contexts [27]. The core elements of the framework are facilitation, innovation, recipients, and context [27]. The identification and integration of the core elements of our strategies are presented in Table 2.

**Figure 1:**
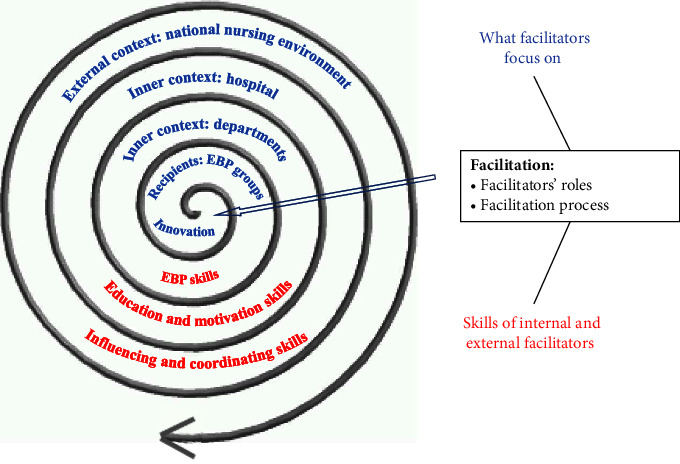
Facilitation process of the EBP-facilitating strategy.

The recipients of this project were the nurses who formed several EBP groups. Initially, six EBP groups, each with a specialty, were set up after an applicant assessment process. Each group elected one person as the leader to run each EBP project on an annual basis. Facilitation is the dynamic component that is involved in evaluating, harmonizing, and merging the other three elements [27]. The facilitation process is shown in Figure 1.

As the internal facilitators, the core team of the nursing department knows the level of EBP skills among the nurses. They invited experts from the Fudan University JBI Evidence-based Nursing Cooperation Center as external facilitators, which provided a structured training program and oversight of the conduct of each EBP project. The first training program involved education and assignments that involved choosing and identifying an EBP problem, reporting the retrieval results, evaluating the quality of the included literature, conducting a meta-analysis, and developing a plan of evidence application project. The training had 32 hours and lasted for two months, and the projects were evaluated at the end of the year. During the second year, the EBP groups continued to conduct new EBP projects.

The core team influenced and coordinated the process, including making decisions to increase or adjust the members and number of groups according to the qualifications of the members and groups. The coordinator updated the group membership annually and arranged for continuous training for new members by an external EBP training program or internal training by experienced members. The training for the new members had four aspects, including identification of an EBP problem, database retrieval, evaluation of the quality of the literature, and the process of evidence application. The training usually was conducted in the first quarter of the year. In addition to the training, the core team organized at least three meetings to assess the process of the EBP projects each year, including the choice of EBP projects, and engaged in ongoing assessment and with the conclusion of the projects. The external facilitator attended meetings more frequently during the first several years until the internal facilitators were sufficiently experienced and groups had gained more experience in implementation of evidence. From 2020, the practice of the strategy entered the sustained phase, the number of EBP group members increased considerably, as the core team planned to extend the activities of EBP throughout the hospital. Then, a second structured training program was held, for which the main focus of the training was the application of evidence.

### 2.4. Data Collection

The validated Chinese version of the self-report Evidence-Based Practice Questionnaire (EBPQ) was used to assess the attitudes, knowledge, and practice of the group members of the EBP projects and general nurses in the same hospital.

The EBPQ was developed by Upton and Upton [29] and was translated into Chinese by Yang and Tang in 2009 [20]. The Chinese version of the EBPQ consists of 24 items allocated to three subscales: knowledge/skills (14 items), attitudes (4 items), and practice (6 items). Each item is rated from 1 to 7, with a higher score as indicating more knowledge, a more positive attitude, or better utilization in regard to EBP. Cronbach's alpha of the Chinese version was 0.94, and the subscales' values were 0.79 to 0.94 [20]. The response to each item was deemed positive if the score exceeded 4. For data analysis, we applied the means of the scores of each subscale and the total EBPQ score. The Chinese version of EBPQ was obtained from the authors for use.

The first survey was administered to EBP group members through a manual questionnaire during February 2017 (T1) after the EBP-facilitating strategy had been applied for one year. The same questionnaire was also administered to general nurses who attended in-service training classes within the hospital. To ensure timely and efficient data collection, we employed convenience sampling to select four classes that were held for different levels of nurses. This method allowed us to readily access a pool of nurses with varying levels of expertise who were available and willing to participate in the study. The second survey was electronic, administered to EBP group members during December 2019 (T2), when the EBP-facilitating strategy had been applied for four years.

During the ongoing assessment and for the concluding report of the EBP projects each year, the core team of the nursing department assessed the results and used a self-designed form to create a record of the project. The form included the information on the project, whether the evidence was applied to clinical practice, and the reasons for the lack of success, if relevant, of the application. The information included the title of the project, group name, setting and population, and main clinical and academic outcomes (e.g., whether a paper was published, whether expert consensus was achieved). The main reasons for a lack of success included insufficiency of the evidence, small gap between current practice and evidence, stakeholder conflicts, and insufficient leadership during evidence implementation. Two members of the core team determined the reasons after discussion with the leader of each EBP group.

### 2.5. Data Analysis

The scores for the knowledge/skills, attitudes, and practice subscales were continuous variables, which were presented as means and standard deviations (SD). The differences between the two groups and the two time points were analyzed using an unpaired Student's *t*-test. A two-tailed *p* value of <0.05 was considered statistically significant. The analyses were performed using SPSS 24.0.

## 3. Results

The mean scores of the total EBPQ were 4.46 (SD = 0.92) after the training and one year of the EBP-facilitated program. The scores for knowledge, attitudes, and practice of the EBP group members were higher than those of general nurses (all *ps* < 0.05). Among EBP members, the scores for knowledge, attitudes, and practice increased after four years of the program (T2), and the differences were significant (all *ps* < 0.05).  Table 3 presents these results.

EBP groups conducted 51 EBP projects that covered emergency nursing, surgical and medical nursing, critical care, nursing management, and so forth. Among the projects, 33 were found to improve nursing practice, as seen in changes of nursing procedures, upgrading of tools, development of nursing standards, and improvement in patient outcomes. For example, the emergency group formulated an EBP for rewarming for patients with traumatic hypothermia, including developing the warming-starting standards, clinical warming methods, and monitoring frequency. A total of 41 patients with traumatic hypothermia participated in the nursing program. The mean temperature of patients was (36.31 ± 1.12) °C when leaving emergency room, and the increase in patients' temperature was (0.82 ± 0.56) °C, which was higher than that before the EBP program (*p* < 0.05). In addition, the percentage of patients with chills was 2.44%, which is significantly lower (*p* < 0.001). Finally, 18 projects were not successful. The main reasons included stakeholder conflicts (6 projects), insufficient leadership during evidence implementation (5 projects), a small gap between current practice and evidence (5 projects), and a lack of evidence (2 projects).

## 4. Discussion

In our study, we employed the i-PARIHS framework to guide an organizational-level EBP-facilitation approach that used an EBP group-based and project-oriented strategy to improve evidence-based practice among nurses in a tertiary general hospital. The results of the first survey indicated that the EBP group members generally viewed EBP positively and had higher knowledge/skills, and practice levels than did general nurses. The baseline of the two groups differed, but the results indicated that the EBP group members had higher skills and a better attitude. The use of intermittent training of EBP, similar to a 30-hour EBP training intervention, was effective in improving the knowledge, attitude, practice, and competency of EBP among nurse educators [30]. The results of the second survey showed that the knowledge, attitudes, and practice of the EBP group members had an upward trend. A systematic review showed that the most frequent types of knowledge transition interventions implemented were educational and interactive, which were more effective than was didactic (Forsetlund et al., 2009; [12]). In our study, the interaction within the group during the implementation of the project as well as between the teachers (external facilitators) and students (EBP group members) explained the large effect in regard to improving knowledge, attitudes, and the practice level of nurses.

The i-PARIHS framework offers a structured approach for the implementation of EBP, allowing researchers to systematically consider all key factors that influence implementation, including evidence, context, and facilitation. In our EBP-facilitating strategy, the composition of the EBP group, roles of internal and external facilitators, and annual audit of EBP projects were the key components.

Lack of academic preparation is one of the barriers to EBP; appropriate composition of and training courses for groups, however, can address this barrier. Nurses with a master's degree had a better understanding of EBP and the associated skills and were able to help retrieve and synthesize the evidence that is the basis of the project. This is the reason that we utilized a high percentage of nurses with a master's degree in the groups. The leader of the group is crucial during the project; thus, a head nurse or supervisor was appointed as the leader because such an individual possesses the type of leadership that enables successful implementation. A nurse manager (head nurse or supervisor) has a particularly influential role in the implementation of EBP in terms of providing a supportive culture and environment [31–33]. The lack of sufficient leadership during evidence implementation can lead to failure, which is what occurred in five projects.

The role of facilitation in the process of implementation is a key factor in enabling the successful implementation of our EBP-facilitating strategy. The core team of the nursing department worked as internal facilitators, making the process smoother by motivating the EBP groups and continuously monitoring the process of the EBP projects. External facilitators were outside experts who brought specialized knowledge and a fresh perspective. As internal resources were insufficient for EBP, these external facilitators trained internal ones and provided ongoing systematic training and guidance during each project.

The ongoing projects, conducted each year, motivated EBP group members to use research evidence and change nursing practice. As noted in the second survey, a prominent upward trend was observed in the knowledge, attitudes, and practices of EBP group members. Despite 18 projects that failed to effectuate a change in practice, members of the EBP group acquired valuable experience, contributing to their growth in the domain of evidence-based practice. Of the projects undertaken, 33 demonstrated significant improvements in nursing practice. These enhancements included modifications to nursing procedures, advancement of tools, development of nursing standards, and enhancement of patient outcomes. This is in keeping with the primary objective of this study, as evidenced by these EBP projects, which was to augment the efficacy of our EBP-facilitation strategy.

### 4.1. Limitations

The main limitation of this study was that the design was a one-arm study, with the lack of a comparison study to evaluate the effect of this group-based and project-oriented EBP strategy. Nevertheless, we observed the effect of the strategy from a longitudinal perspective (over six years) and found the strategy suitable for the culture and characteristics of our hospital. Moreover, the strategy can be applied to similar settings. Finally, had we assessed the knowledge, attitudes, and practice of the nurses each year, our findings would have been more rigorous.

## 5. Conclusion

Earlier research typically focused on one targeted clinical problem or on changing nursing behavior, with limited focus on knowledge translation interventions or strategy from an organizational perspective. We developed a group-based and project-oriented EBP strategy, under the guidance of the i-PARIHS framework that can promote the knowledge and attitudes of nurses and increase their level of practice, thus improving patients' outcomes. The composition of the EBP group, annual audits of EBP projects, and the roles of internal and external facilitators were key components of this EBP-facilitating program.

## 6. Implications for Nursing Managers

Enhancing EBP among nurses in general hospitals is critical to nursing quality, but there are still shortfalls in practice, especially in under-resourced countries. The i-PARIHS framework provides a theoretical framework for the development of an organizational-level EBP-facilitation approach based on the context of the organization. This enriches the application of the i-PARIHS framework. An EBP group-based and project-oriented strategy can improve certain nurses' academic preparation for EBP through ongoing training and sustained EBP behavior that involves annual projects. Nursing research should continue to analyze the results of projects and the characteristics of EBP behavior to promote EBP.

## Figures and Tables

**Table 1:** Number of groups and nurses in each year.

Year	Groups (*n*)	Nurses (*n*)	Head nurse/supervisor (*n*, %)	Master's degree (*n*, %)
2016	6	34	13 (38.24)	13 (38.24)
2017	8	49	20 (40.82)	16 (32.65)
2018	8	54	17 (31.48)	26 (48.15)
2019	8	56	20 (35.71)	29 (51.79)
2020	10	83	28 (33.73)	35 (42.17)
2021	11	96	28 (29.17)	46 (47.92)

**Table 2:** Identification of core elements in the EBP-facilitating strategy.

Core element	Strategy
Innovation	Enhance the evidence-based practice skills of nurses
	Promote the application of research evidence to inform the innovation

Recipients	EBP groups formation process: (a) The core team developed the recruitment criteria
	Recruitment criteria: committed to EBP; have skills related to EBP; encourage master's degree and head nurses to participate (b) Nurses submitted the application to nursing department
	(c) The core team assessed the applicants and set up EBP groups. Principles: each group had nurses from the same or related specialties and comprised nurses in a leadership position (head nurse/supervisor/vice director) and who had a master's degree

Context	Local level
	Same or related specialties had a similar setting and environment, which ensured the same interest in choosing EBP questions and enabled the implementation of research evidence
	Organization level
	Hospital and nursing department supported and provided training, multidiscipline collaboration, and necessary facilities
	External healthcare system level
	The priority was to narrow the gap between evidence and practice and improve patients' outcomes; this was hampered, however, by a lack of EBP skills

Facilitation	Facilitators
	Internal facilitator: the core team of nursing department; one member was assigned as the coordinator of this project
	External facilitator: three members of the Fudan University JBI Evidence-based Nursing Cooperation Center
	Facilitation strategies
	The core team organized a two-month intermittent training of EBP guided by a well-educated EBP team
	Each EBP group was required to conduct one EBP project per year. Regular meetings were held for audits of the projects
	The coordinator tracked the process of each EBP project and provided regular feedback
	The coordinator reviewed and updated the members of EBP groups annually
	An external facilitator navigated the implementation of evidence in projects

**Table 3:** Comparison of nurses' EBP knowledge/skills, attitudes, and practice (mean ± SD).

Group	*n*	Knowledge/skills	Attitudes	Practice	Total
EBP members	40	4.31 ± 0.99	5.38 ± 0.90	4.20 ± 1 0.14	4.46 ± 0.92
General nurses	152	3.85 ± 0.75	4.64 ± 1.00	3.84 ± 0.91	4.02 ± 0.69
*t* value		2.745	4.185	2.115	3.769
*p* value		0.008	<0.001	0.036	<0.001
EBP members (T1)	40	4.31 ± 0.99	5.38 ± 0.90	4.20 ± 1.14	4.46 ± 0.92
EBP members (T2)	48	4.74 ± 0.97	5.80 ± 0.81	4.96 ± 1.21	4.97 ± 0.89
*t* value		−2.072	−2.299	−3.010	−2.649
*p* value		0.041	0.024	0.003	0.010

## Data Availability

The data used to support the findings of this study are available from the corresponding author upon request.
